# Regulation of Differentiation by Calcium-Sensing Receptor in Normal and Tumoral Developing Nervous System

**DOI:** 10.3389/fphys.2016.00169

**Published:** 2016-05-10

**Authors:** Silvia Mateo-Lozano, Marta García, Carlos J. Rodríguez-Hernández, Carmen de Torres

**Affiliations:** ^1^Developmental Tumor Biology Laboratory, Institut de Recerca Pediàtrica – Hospital Sant Joan de DéuBarcelona, Spain; ^2^Department of Oncology, Institut de Recerca Pediàtrica – Hospital Sant Joan de DéuBarcelona, Spain

**Keywords:** development, nervous system, neuroblastoma, calcium-sensing receptor, differentiation, differentiation and proliferation

## Abstract

During normal development of the nervous system (NS), neural progenitor cells (NPCs) produce specialized populations of neurons and glial cells upon cell fate restriction and terminal differentiation. These sequential processes require the dynamic regulation of thousands of genes. The calcium-sensing receptor (CaSR) is temporally and spatially regulated in both neurons and glial cells during development of the NS. In particular, CaSR expression and function have been shown to play a significant role during differentiation of NPCs toward the oligodendrocyte lineage and also in maturation of cerebellar granule cell precursors (GCPs). Moreover, CaSR regulates axonal and dendritic growth in both central and peripheral nervous systems (PNSs), a process necessary for proper construction of mature neuronal networks. On the other hand, several lines of evidence support a role for CaSR in promotion of cell differentiation and inhibition of proliferation in neuroblastoma, a tumor arising from precursor cells of developing PNS. Thus, among the variety of NS functions in which the CaSR participates, this mini-review focuses on its role in differentiation of normal and tumoral cells. Current knowledge of the mechanisms responsible for CaSR regulation and function in these contexts is also discussed, together with the therapeutic opportunities provided by CaSR allosteric modulators.

The calcium-sensing receptor (CaSR), a G protein-coupled receptor (GPCR) whose primary ligand is calcium, was initially identified in the parathyroid gland, where it regulates calcium homeostasis (Brown et al., 1993). Subsequent studies found that this GPCR is present in many other organs (Riccardi and Kemp, 2012) and in some cancers (Brennan et al., 2013), where it plays versatile roles. CaSR also participates in a wide range of cellular functions that are important for proper development of the nervous system (NS; Bandyopadhyay et al., 2010; Ruat and Traiffort, 2013), and any alterations in its expression and/or function may lead to disease, including tumors.

During development, cell fate restriction occurs in the NS where specialized neuronal and glial populations arise from neural progenitor cells (NPCs). This is achieved without changes of DNA sequence through the coordinated regulation of gene expression promoted by cell-intrinsic programs, such as epigenetic mechanisms and transcription factors, as well as extracellular cues. Increasing evidence indicates that proper CaSR expression and function is important during differentiation of specific neural precursor cells upon commitment toward neuronal and glial fates. CaSR also plays significant roles during differentiation of neuroblastoma, a developmental tumor of the peripheral nervous system (PNS). Therefore, among many other functions of the NS in which it participates (Bandyopadhyay et al., 2010; Ruat and Traiffort, 2013), this mini-review focuses on the role of CaSR in differentiation, a cellular process that is crucial for both normal development and tumor biology.

## Role of CaSR in differentiation of normal developing nervous system

The rat *Casr* gene was cloned from a striatal cDNA library by homology screening (Ruat et al., 1995). Subsequent expression analyses identified its presence in almost all regions of the central nervous system (CNS) including hypothalamus, striatum, hippocampus, pituitary, cerebellum, brainstem, circumventricular organs, and spinal cord (Ruat et al., 1995; Chattopadhyay et al., 1997; Rogers et al., 1997).

The CNS is composed of neurons and three major populations of glial cells, astrocytes, oligodendrocytes, and microglia. Although, expression of CaSR has been reported in astrocytes (Chattopadhyay et al., 2000; Dal Pra et al., 2005) and microglia (Chattopadhyay et al., 1999a), the CaSR was first localized to nerve terminals of neurons and fiber tracts (Ruat et al., 1995). Nerve tracts consist of axons wrapped by myelin sheaths which are produced by oligodendrocytes in the CNS and by Schwann cells in the PNS. In order to be able to produce myelin, oligodendrocyes precursor cells (OPCs) progress through a series of differentiation steps, lose their capacity to proliferate and migrate, and finally generate mature oligodendrocytes (Barateiro and Fernandes, 2014).

Several lines of evidence support a role for CaSR in this differentiation process. First, a period of increased CaSR expression was identified during rat postnatal development in myelinated structures (Chattopadhyay et al., 1998; Ferry et al., 2000). By double *in situ* hybridization, *Casr* and myelin basic protein (*Mbp*) mRNAs were shown to co-localize in cerebellum, brainstem, corpus callosum, fimbria of the hippocampus, *stria medullaris*, and lateral olfactory tracts during myelogenesis. Second, Northern blot and reverse transcription polymerase chain reaction (RT-PCR) confirmed *Casr* mRNA expression in purified oligodendrocytes. Moreover, exposure to high ${\text{Ca}}_{\text{o}}^{2+}$ and calcimimetic NPS R-568 resulted in phosphatidylinositol hydrolysis and intracellular Ca^2+^ (${\text{Ca}}_{\text{i}}^{2+}$) mobilization (Chattopadhyay et al., 1998; Ferry et al., 2000). Altogether, these data indicate that a functional CaSR is present in olygodendrocytes and temporally regulated during OPCs differentiation.

The role of CaSR in the transition of neural precursor cells toward the oligodendrocyte lineage has also been established (Chattopadhyay et al., 2008). To this end, neural stem cells were isolated from fetal rat brains and induced to commit to neuronal, oligodendrocyte, or astrocytic lineages. *Casr* expression increased in OPCs, remained high during the premyelinating stage and declined in mature oligodendrocytes. Furthermore, *Mbp* mRNA levels increased in OPCs exposed to high ${\text{Ca}}_{\text{o}}^{2+}$ or spermidine for 1–3 days. This phenotype was blocked by overexpression of a naturally-occurring dominant-negative CaSR variant p.Arg185Gln (Bai et al., 1997). Furthermore, *Mbp* mRNA levels were significantly reduced in the cerebellum of 2-week old *Casr*-null (*Casr*^−∕−^) mice as compared to age-matched *Casr*^+∕+^ mice. Altogether, these results indicate that prolonged CaSR activation promotes maturation of OPCs. However, a brief exposure to high ${\text{Ca}}_{\text{o}}^{2+}$ induced OPCs proliferation, suggesting that acute and long-term activation of CaSR differentially affects cell proliferation and differentiation (Chattopadhyay et al., 2008).

Studies conducted in *Casr*^−∕−^ mice (Liu et al., 2013), a mouse model of human neonatal severe hyperparathyroidism (Ho et al., 1995), have provided direct *in vivo* evidence for CaSR roles during differentiation of CNS. In these mice, both brain weight and size were reported to be lower than that of wild-type littermates during the first 2 weeks of postnatal development. Small brain size was associated with a reduced number of cells and proliferation rates, but deletion of the parathyroid hormone (*Pth*) gene, which corrects hyperparathyroidism, hypercalcemia, and hypophosphatemia, normalized these alterations. Interestingly, decreased expression of neuronal (neuronal nuclear antigen, NeuN) and glial (glial fibrillary acidic protein and MBP) differentiation markers were detected in these brains, and levels of expression were not normalized upon deletion of the *Pth* gene, thus suggesting that CaSR is necessary for differentiation of neural progenitors toward neuronal and glial fates, but not for their proliferation.

More recently, the role of CaSR has also been evaluated in the developing cerebellum (Tharmalingam et al., 2016) during a period that includes initial proliferation of granule cell precursors (GCPs) in the external granule cell layer (EGL) followed by differentiation and cell cycle exit. At later stages, differentiated GCPs migrate within the EGL, a process called tangential migration, and then toward the internal granule cell layer (IGL) by radial migration. Immunoblots showed high rat Casr protein expression in the cerebellum from P7 to P18, a period during which maximal GCPs migration occurs. Moreover, CaSR allosteric activators NPS R-568 and R-467 increased GCPs migration *in vitro*, and these effects were blocked by calcilytic NPS 2143 (Bandyopadhyay et al., 2010). Also, calcimimetics promoted increased radial migration of GCPs from the EGL into the IGL. Specificity of this phenotype was corroborated by experiments conducted with NPS 2143. Interestingly, the number of cells positive for NeuN was higher in rats treated with CaSR allosteric activators and reduced in those receiving NPS 2143 when compared to controls. Moreover, rats exposed to the calcilytic also showed significantly increased numbers of Ki67-positive GCPs, a nuclear marker of cell proliferation. Together, these studies argue that CaSR expression and function are necessary for proper migration and differentiation of GCPs.

While several studies have analyzed the expression and function of CaSR in the CNS, much less attention has been devoted to the role of this receptor in the PNS. Several lines of evidence support that CaSR expression and function during normal formation of the PNS are critical for axonal and dendritic growth. Proper regulation of axon growth and branching are crucial for constructing functional, mature neuronal networks. These processes are regulated by extracellular cues, growth factors, and morphogens that signal through receptors, activate intracellular signaling cascades and regulate cytoskeletal dynamics (Kalil and Dent, 2014). Compelling data reported by Vizard et al. (2008) support that CaSR would be among receptors that integrate extracellular signals during axon growth and branching. These authors showed that a brief period of increased *Casr* mRNA expression occurs in mouse neurons of the superior cervical ganglion (SCG) from embryonic day 16 (E16) until E18, a time when murine sympathetic axon are branching at their targets. They functionally showed that neurons at this peak of *Casr* expression display enhanced axonal growth when exposed to high ${\text{Ca}}_{\text{o}}^{2+}$ and calcimimetic NPS R-467, whereas this output is blunted by blocking CaSR function by either calcilytic NPS 89636, *Casr* deletion, or overexpression of a dominant-negative CaSR (Bai et al., 1997). Also, a significant reduction in the iris sympathetic innervation density was shown in *Casr*^−∕−^ mice. Furthermore, a similar role was demonstrated in hippocampal pyramidal neurons, thus providing evidence for a CaSR role in neuronal growth and branching in both PNS and CNS.

## Role of CaSR in differentiation of tumoral developing nervous system

Childhood solid tumors have been recognized as a group of cancers significantly different from adult neoplasias (Scotting et al., 2005; Marshall et al., 2014). They arise from precursor cells during organogenesis and retain many of the morphological and biological features of their undifferentiated, highly proliferative, and sometimes migratory normal cells of origin.

Neuroblastomas originate from PNS precursor cells (Cheung and Dyer, 2013) which in turn derive from trunk neural crest cells. This is a transient population of embryonic cells (LeDouarin, 1982) that generate several derivatives including neurons and glia of the sympathetic NS (Bronner and LeDouarin, 2012). This process involves a period of cellular proliferation, followed by delamination, migration, specification, and terminal differentiation. To produce glial cells and neurons, a portion of trunk neural crest cells migrate along a ventral pathway (Henion and Weston, 1997). Several environmental cues contribute to their fate restriction and, upon terminal differentiation, they give rise to the sympathetic ganglia and medullary region of the adrenal gland (Anderson and Axel, 1986; Anderson et al., 1991).

The potential origin of neuroblastomas in neural crest precursor cells “blocked” at different stages of this process, as well as a combination of various molecular and genetic events, is thought to underlie the heterogeneity of this group of tumors that include both benign and malignant cases (Brodeur, 2003; Maris, 2010; van Noesel, 2012). The most relevant factors associated with these different clinical behaviors of neuroblastomas are age at diagnosis, clinical stage, *MYCN* amplification, alterations of ploidy, numerical and structural chromosomal abnormalities, and histological degree of differentiation (Ambros et al., 1996; Bown et al., 1999; Janoueix-Lerosey et al., 2008; Molenaar et al., 2012; Cheung and Dyer, 2013; Pugh et al., 2013).

The first genetic alteration to be described in neuroblastoma was the amplification of the oncogene *MYCN* (Schwab et al., 1983). It is present in only 22% of these tumors but it is the most significant genetic predictor of poor outcome (Brodeur et al., 1984). MYCN is part of the basic helix-loop-helix family of transcription factors that also includes MYC (c-MYC) and MYCL (Zimmerman et al., 1986; Gustafson and Weiss, 2010). MYCN is a critical promoter of cell proliferation while inhibiting differentiation and apoptosis in early post-migratory neural crest cells and also during CNS neurogenesis (Knoepfler et al., 2002). This is achieved by a complex network of interactions with other transcription factors and epigenetic mechanisms that cooperate to regulate a wide array of genes (Huang and Weiss, 2013). Intriguingly, MYC, which exhibits some structural and functional similarities with MYCN, is an important transcriptional regulator in the transition from proliferating to differentiating OPCs (Magri et al., 2014).

Neuroblastic tumors are composed of two main cellular components: neuroblasts, of neuronal origin, and glial, Schwannian-like cells. Classifications based on the degree of neuroblasts maturation and the extent of the glial component showed that differentiated tumors were associated with good clinical outcome (Hughes et al., 1974; Shimada et al., 1999, 2001). A variety of proteins participate in the differentiation processes of neuroblastomas (reviewed in Mohlin et al., 2011). Among them, the CaSR was found to be highly expressed in differentiated neuroblastic tumors and up-regulated upon differentiation induction (de Torres et al., 2009). CaSR was previously identified in adult CNS tumors (Chattopadhyay et al., 1999b, 2000), but it had not been reported in any developmental malignancy. In neuroblastoma, *CaSR* mRNA expression significantly correlated with several factors associated with good outcome such as age at diagnosis <1 year, low clinical stage and differentiated histology. Immunohistochemistry showed that undifferentiated neuroblasts were mostly CaSR-negative while even the earliest stages of neuroblast differentiation displayed CaSR immunostaining. When present, glial cells were also strongly positive for CaSR. Moreover, upon neuroblastoma differentiation induction, increased CaSR expression was seen both in clinical specimens obtained after treatment, and *in vitro*, at early phases of neuronal differentiation induced by retinoic acid.

In accordance with these data, *CASR* gene silencing by epigenetic mechanisms was found in undifferentiated, *MYCN*-amplified, aggressive neuroblastomas (Casalà et al., 2013). These mechanisms included promoter 2 hypermethylation and histone modifications. CpG islands are clusters of GC dinucleotides located in promoter regions, and also in intragenic regions, that are usually unmethylated (Deaton and Bird, 2011). Methylation of the fifth position of cytosines at CpG islands around promoter regions is a mechanism of gene silencing that inactivates tumor-suppressor genes (Herman et al., 1996; Timp and Feinberg, 2013) and also contributes to gene modulation during cell type specification and lineage commitment (Hirabayashi and Gotoh, 2010; Hu et al., 2014). A particular region of the CpG island encompassing *CASR* gene promoter 2 was found to be hypermethylated in 25% primary neuroblastomas, in association with reduced *CaSR* mRNA expression, *MYCN* amplification, undifferentiated histopathology and other factors of poor outcome. In neuroblastoma cell lines, treatment with demethylating agent 5′aza-2-deoxycitidine and/or histone deacetylase inhibitor trichostatin A decreased the percentage of methylated cytosines in this specific region of *CASR* gene promoter and concomitantly restored *CaSR* expression in *MYCN*-amplified cell lines. Association of *MYCN* amplification and epigenetic silencing of several genes in neuroblastoma had been previously reported (Alaminos et al., 2004), although the underlying mechanisms are still under investigation (Perini et al., 2005; Hervouet et al., 2009; Murphy et al., 2009, 2011; He et al., 2013). In addition, monosomy of chromosome 3, where the human *CASR* gene resides, was observed in >90% of primary neuroblastic tumors of all subgroups by interphase fluorescence *in situ* hybridization. Interestingly, other genes that exert tumor-suppressor functions in neuroblastoma, like *RASSF1A*, are also located on chromosome 3 and hypermethylated in neuroblastic tumors and phaeochromocytomas, which are also PNS tumors (Astuti et al., 2001). Furthermore, ectopic overexpression of full-length CaSR in two *MYCN-*amplified cell lines in which this gene was previously shown to be silenced by promoter hypermethylation significantly decreased their proliferative and tumorigenic capacities. Moreover, acute exposure to high ${\text{Ca}}_{\text{o}}^{2+}$ concentrations prompted their apoptosis. In all, these data provided functional evidence of the biological relevance of CaSR epigenetic silencing in neuroblastoma biology.

In addition, when non-synonimous genetic variants located at the intracellular tail encoded by exon 7 of the *CASR* gene were analyzed in a cohort of neuroblastoma patients, a haplotype including a polymorphism considered to mildly reduce CaSR activity (Heath et al., 1996; Cole et al., 1999; Scillitani et al., 2004, 2007; Hu and Spiegel, 2007; Vezzoli et al., 2007; Yun et al., 2007) was associated with poor outcome (Masvidal et al., 2013).

**Figure 1:**
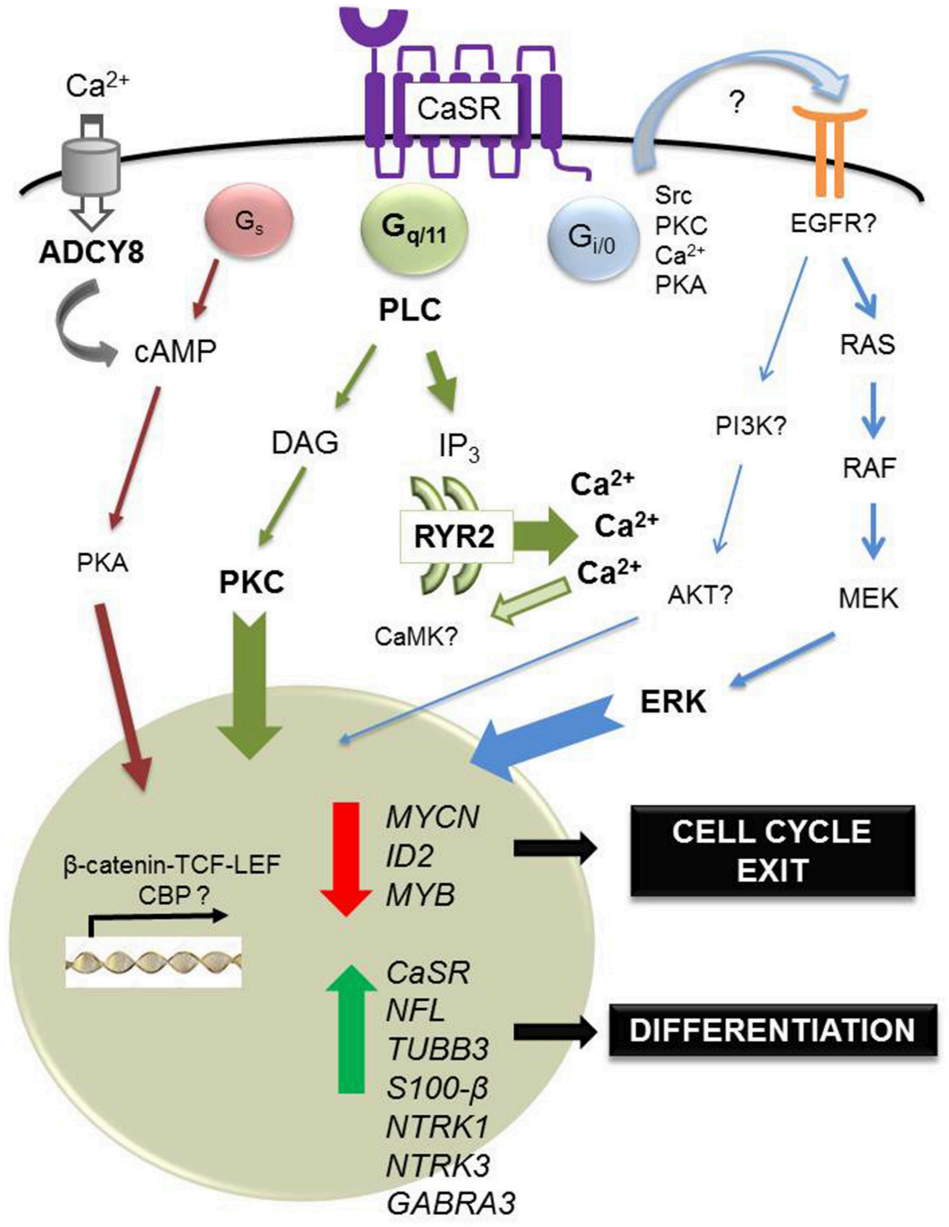
**Regulation of differentiation by calcium-sensing receptor in neuroblastoma**. In neuroblastoma, the CaSR is expressed in benign, differentiated tumors. In this tumoral context, the main physiological ligand remains unknown. However, acute exposure to high extracellular concentrations of Ca^2+^ induces apoptosis of CaSR-positive, *MYCN-*amplified cells, dependent on sustained activation of ERK. Also, short *in vitro* exposure to cinacalcet, an allosteric activator of the CaSR approved for clinical use, induces endoplasmic reticulum (ER) stress coupled to apoptosis in CaSR-positive, *MYCN*-amplified neuroblastoma cells. This output is dependent on activation of phospholipase C (PLC). Massive Ca^2+^ exit from the ER up-regulates ryanodine receptor 2 (*RYR2*) and activation of adenylyl cyclase type 8 (ADCY8) via capacitative calcium entry, a mechanism triggered by depletion of intracellular Ca^2+^ stores. Furthermore, prolonged treatment with cinacalcet promotes up-regulation of genes associated with neuroblastoma differentiation (*NFL, TUBB3, S100-*β, *NTRK1, NTRK3, GABRA3*) and down-regulation of genes that are critical for proliferation of these tumors (*MYCN, ID2, MYB*). Concomitantly, sustained exposure to cinacalcet also induces up-regulation of CaSR and increased expression of cancer-testis antigens.

Finally, a recent study has shown that cinacalcet, an allosteric activator of the CaSR approved for clinical use (Nemeth et al., 1998), inhibits neuroblastoma tumor growth *in vitro* and *in vivo* (Rodríguez-Hernández et al., 2016). Mechanisms involved include ER stress coupled to apoptosis dependent on phospholipase C activation in *MYCN*-amplified neuroblastoma cells and, irrespective of *MYCN* status, differentiation of surviving cells. Induction of differentiation was also observed upon prolonged exposure to cinacalcet *in vivo.* Genome-wide gene expression analysis by microarrays of xenografts showed up-regulation of numerous genes involved in neuroblastoma differentiation, like *NTRK3* (Nakagawara et al., 1993) and *GABRA3* (Roberts et al., 2004). Gene Ontology categories also unveiled up-regulation of genes involved in axon growth like doublecortin and ephrins (Kalil and Dent, 2014). Concomitantly, genes that critically support neuroblastoma proliferation were down-regulated, such as *MYCN*, inhibitor of differentiation 2 (*ID2*) and *MYB* (Figure 1). ID2 is a helix-loop-helix transcription factor controlled by MYC proteins that blocks differentiation and promotes cell proliferation (Lasorella et al., 2000), and MYB is a transcription factor that cooperates with MYCN in cell cycle regulation of *MYCN*-amplified neuroblastomas (Gualdrini et al., 2010). Quite unexpectedly, cinacalcet also promoted up-regulation of cancer-testis antigens, a family of proteins that are almost exclusively expressed in tumor cells and are thus considered ideal targets for immunotherapy (Fratta et al., 2011). Although mechanisms involved are not yet understood, histone acetyltransferases such as p300 and CREB binding protein might be recruited, taking into account that these genes are mainly regulated by epigenetic mechanisms (Rao et al., 2011).

In summary, studies conducted in neuroblastoma indicate that CaSR promotes differentiation and inhibits proliferation in this malignancy. Also, they are in accordance with data obtained in developing NS supporting that CaSR plays a significant role in differentiation processes of specific NPCs upon commitment toward neuronal and glial lineages. More importantly, neuroblastoma models show that pharmacological modulation of CaSR activity can provide novel therapeutic opportunities.

## Conclusions and future directions

Over the past decades, the patterns of expression of CaSR in the NS have been described. However, our understanding of CaSR regulation and functions during normal and pathological development of CNS and PNS is incomplete. In the coming years, epigenetic mechanisms responsible for CaSR regulation during formation of NS will be elucidated. They will probably involve cytosines methylation and demethylation by DNA methyltransferases and 10–11 translocation enzymes (Hahn et al., 2013), histones modifications and non-coding RNAs. Also, the complex interplay of these mechanisms with transcription factors such as the MYC family will be characterized. This knowledge, together with a precise picture of signaling pathways downstream of CaSR during differentiation processes, will help to evaluate whether pharmacological modulation of this GPCR might be beneficial in the treatment of NS developmental diseases.

## Author contributions

CdT is the Principal Investigator of the project. SM-L, CR-H, and MG are postdoctoral researchers working in the project, notably contributing to the development of both execution and conception of experiments.

### Conflict of interest statement

CdT holds a patent, WO 2013144397 (A1), and a patent submission (PCT/ES2015/070561) on cinacalcet. The other authors declare that the research was conducted in the absence of any commercial or financial relationships that could be construed as a potential conflict of interest.
